# The Diagnostic Significance of Usual Biochemical Parameters in Coronavirus Disease 19 (COVID-19): Albumin to Globulin Ratio and CRP to Albumin Ratio

**DOI:** 10.3389/fmed.2020.566591

**Published:** 2020-11-03

**Authors:** Gavriela M. Feketea, Vasiliki Vlacha

**Affiliations:** ^1^Department of Hematology, “Iuliu Hatieganu” University of Medicine and Pharmacy, Cluj-Napoca, Romania; ^2^Department of Pediatrics, Pediatric Allergy Outpatient Clinic, Karamandaneio Children Hospital of Patras, Patras, Greece; ^3^Department of Early Years Learning and Care, University of Ioannina, Ioannina, Greece

**Keywords:** albumin/globulin ratio (AGR), CRP/albumin ratio, COVID-19, SARS-CoV-2, kawasaki like disease, ARSD, ICU - intensive care unit

The clinical course of patients with COVID-19, and the progression to severe disease, are difficult to predict. There is a growing interest in identifying individuals who could be at greater risk for developing severe or critical COVID-19, acute respiratory distress syndrome (ARDS) in adults, Kawasaki-like disease in young individuals and children, and even death. Several laboratory parameters have been analyzed in conjunction with COVID-19. Specifically, significant elevation of C-reactive proteins, erythrocyte sedimentation rate, interleukin-6, and lactate dehydrogenase has been observed. On the other hand, the total white blood cell count and the eosinophilic and lymphocytic count have been decreased in COVID-19 patients. In a recent meta-analysis, increased CRP, lymphopenia, and increased LDH were significantly associated with the severity of the disease (1). The levels of certain laboratory values that proved to be elevated in cytokine storm (ferritin, procalcitonin, and troponin) may not be available at most hospital laboratories or are mainly used for research purposes (IL-6) (2, 3). On the other hand, CRP, albumin, and globulin are readily available, shortly after admittance, and are often part of an admission workup, in general hospitals and particularly in intensive care units (ICU).

Albumin and globulin are two important components of serum proteins and have been proven to be involved in systemic inflammation. A low serum albumin reflects a poor nutritional status, liver and kidney dysfunction, and has been shown to be an independent predictor of poor survival in critically ill patients. Furthermore, similar results were found regarding MERS (4). Decreased albumin at admission has been an independent risk factor associated with unimprovement during follow-up in COVID-19 patients (5). On the other hand, an increased globulin level may reflect a chronic inflammatory response. Thus, the additive effect of both albumin and globulin would not only be a prognostic factor for potential COVID-19 complications during the course of the illness, but also an initial risk index of SARS-CoV-2 positive individuals. Wu et al. showed recently that the level of albumin is significantly lower [30.40 g/L (27.15–33.35) vs. 33.70 g/L (30.95–36.30), *p* < 0.001] and the globulin level higher [31.60 g/L (29.35–35.05) vs. 30.00 g/L (28.25–32.55), *p* = 0.004] in COVID-19 patients with ARDS comparative with those without ARDS (6).

The albumin to globulin ratio (AGR) is calculated according to the following formula: AGR = albumin/globulin. In young children, who to date are not at a high risk for developing critical COVID-19, the AGR is higher than in adults due to constitutionally lower globulin. Contrary to this, in hypertension, diabetes, cardiovascular disease, and cancer, which frequently are comorbidities in critical COVID-19, the AGR is lower than normal mainly due to hypoalbuminemia. In different solid tumor patients, a high AGR has a significant positive prognostic effect on survival (7). In patients hospitalized with H_1_N_1_ infection, AGR reversal (ratio <1) was associated with prolonged hospital stay, ICU admission, and ventilator use (8).

Two months after the spread of SARS-CoV-2 in Italy, Verdoni et al. found a 30-fold increased incidence of Kawasaki-like disease in children (9), a rare acute hyper-inflammatory syndrome emerging during the COVID-19 pandemic (10) with coronary artery aneurysms as its main complication. The potential for missed or late diagnosis and consequently delayed treatment of Kawasaki disease in children leading to an increased risk of coronary artery aneurysms, is of particular concern during this pandemic (11). Just before the pandemic, Mammadov et al. using a multivariate analysis found that a lower AGR served as an independent predictor of coronary artery aneurysms in children with Kawasaki disease and had a sensitivity of 56.25% and a specificity of 61.11% at a cutoff value of <1.48 (12).

Older age, chronic lung disease, cardiovascular disease, diabetes mellitus, obesity, immunocompromise, end-stage renal disease, and liver disease have been established as potential risk factors for severe COVID-19 (13). The AGR can be decreased in those conditions. In chronic kidney disease patients, it has been shown that AGR was associated with patient mortality of all causes as well as of cardiovascular diseases (14). Acute liver injury due to COVID-19 is associated with increased death risk, as it has been shown by Fu (15). The total protein, albumin, and albumin/globulin ratio were decreased in critically ill patients with acute liver injury compared with those with a less severe illness. In addition, the albumin levels were lower in patients with concurrent diabetes. In that analysis, it has been also shown that the fatality rate was higher in older patients and in ones with hypoproteinemia. A decreased albumin/globulin ratio has been also identified by Tian et al. as one of the risk factors related with the severity of COVID-19 in patients with cancer (16).

Taking all the above into consideration we believe that to calculate the AGR during a prolonged fever, at admission and also throughout the course of the illness, would be useful as a prognostic index for severe COVID-19 including ARDS and in young individuals and children with Kawasaki-like disease.

C-reactive protein (CRP) induction is part of the acute phase response, in which the synthesis of many plasma proteins is increased, whereas a smaller number, particularly albumin, is decreased (17). It has been reported that the patients who needed invasive ventilation had increased inflammatory markers, including CRP (18). Hypoalbuminemia (5) and elevated CRP (19) has been shown to be associated with severe illness and death in patients with COVID-19. The CRP to albumin ratio has been shown to be more accurate than CRP alone for predicting the 28-day mortality in critically ill patients (20). In addition, a higher CRP to albumin ratio at admission has been determined as a risk factor for in-hospital mortality in elderly patients with acute kidney injury requiring dialysis (21). Similarly, an initial elevated CRP to albumin ratio was significantly related to 28-day mortality associated with infections caused by *Elizabethkingia* spp., a dangerous opportunistic bacterial pathogen causing different illnesses including pneumonia (22). The authors concluded that the prediction of clinical courses using an initial CRP to albumin ratio is a priority to reduce the mortality in these patients. We have calculated the CRP to albumin ratio from the published data of an earlier publication by Liu et al. regarding SARS-CoV-2 infected patients (23). The median (range) of the ratio was 9.6 (1.0–2.5). One patient, who was diagnosed with shock and respiratory failure, had the highest value. It is also interesting to note that the patients with the lowest values were younger and without comorbidities. However, the study was limited to 12 patients.

Xie et al. found that CRP levels had a sensitivity of 65.52% and a specificity of 62.7% for predicting IVIG-resistance at a cutoff point of >100 mg/L in children with Kawasaki disease. In addition, albumin <32 g/L, had a sensitivity and specificity for predicting IVIG-resistance 72 and 83.19%, respectively (24).

The three laboratory values, albumin, globulin, and CRP have been separately reported to be associated with normal aging. Healthy older people have been shown to have low serum levels of CRP and pro-inflammatory cytokines such as IL-6 compared with older people with comorbidities. However, those values are higher in aging people than in the younger population (25). Additionally, the increased levels of those parameters are associated with the raised risk of morbidity and mortality in the older subjects (26).

The albumin serum concentration has been shown to decrease with age (27–30). It has been also demonstrated that the globulin levels are higher in older people compared with younger people (31). Both nutritional and chronic inflammatory status, as it results from different comorbidities and aging, clearly influence the prognostic of COVID-19. In addition, inflammation due to COVID infection is added to this status.

There is an increased urgent need to detect new biomarkers in order to identify cases of COVID-19 that will evolve unfavorably in adults and children. These biomarkers must be easy to measure and accessible to most hospitals that manage COVID-19 cases. The proposed ratios (albumin to globulin and CRP to albumin) seem to be more accurate than each value separately and could be included in the initial assessment of patients that have tested PCR positive for SARS-CoV-2, in order to identify those who are at risk of developing ARDS or Kawasaki disease in young individuals and children. In addition, they can be measured during hospital or ICU admissions to evaluate the course of the illness.

## Author Contributions

GF and VV contributed to the design and implementation of the research, to the analysis of the results, and to the writing of the manuscript. All authors contributed to the article and approved the submitted version.

## Conflict of Interest

The authors declare that the research was conducted in the absence of any commercial or financial relationships that could be construed as a potential conflict of interest.
